# Joint Transmission Power Control and Relay Cooperation for WBAN Systems

**DOI:** 10.3390/s18124283

**Published:** 2018-12-05

**Authors:** Hongyun Zhang, Farzad Safaei, Le Chung Tran

**Affiliations:** 1 Artificial Intelligence Research Center, National Innovation Institute of Defense Technology, Beijing 100071, China; 2 School of Electrical, Computer and Telecommunications Engineering, Faculty of Engineering and Information Science, University of Wollongong, Wollongong, NSW 2522, Australia; farzad@uow.edu.au (F.S.); lctran@uow.edu.au (L.C.T.)

**Keywords:** transmission power control, relay cooperation, dynamic slot scheduling, channel autocorrelation, wireless body area networks, IEEE 802.15.6

## Abstract

Improving transmission reliability is a crucial challenge for Wireless Body Area Networks (WBANs) because of the instability of channel conditions and the stringent Packet Loss Ratio (PLR) requirement for many WBANs applications. On the other hand, limited by the size of WBAN nodes, the energy consumption of WBAN nodes should be minimized. In this paper, we jointly consider transmission power control, dynamic slot scheduling and two-hop cooperative mechanism and propose an Autocorrelation-based Adaptive Transmission (AAT) scheme that achieves a better trade-off between transmission reliability and energy consumption for WBAN systems. The new scheme is designed to be compatible with IEEE 802.15.6. We evaluated the performance of the newly proposed scheme by importing the real channel datasets into our simulation model. Simulation results demonstrate that the AAT method can effectively improve the transmission reliability while reducing the energy consumption. We also provide the performance evaluation from three perspectives, namely packet error ratio, energy consumption and energy efficiency, and provide recommendations on the application of the two-hop cooperative mechanism associated with the proposed AAT in the contexts of WBANs.

## 1. Introduction

Wireless body area networks (WBANs) are radio networks of sensors and/or actuators, placed on, in or around the human body, and represent the latest generation of personal area networks [1]. WBANs can potentially enable a wide range of applications, including healthcare, medical, emergency services, sports, and consumer entertainment. However, WBANs face two main transmission challenges, i.e., transmission reliability and energy efficiency. On the one hand, due to the presence of the human body and the complexity of the surrounding environment, the wireless channels in WBAN exhibit significant peculiarity in comparison with other Wireless Sensor Network (WSN) channels [2,3]. Many factors contribute to the severe signal attenuation (over 100 dB has been observed [4]) of WBAN channels. These factors include diffraction, reflection, energy absorption, antenna losses, shadowing by the body tissue, and body posture.

On the other hand, to ensure the comfortable and unobtrusive deployment of wireless devices in/on the human body, WBAN sensor or actuator nodes are required to have a small size, which obviously limits the battery capacity and the WBAN system lifespan. Meanwhile, in many WBAN applications, changing or replacing the battery is infeasible. For example, a pacemaker or a glucose monitor would require a lifetime lasting more than five years [5]. For this reason, WBAN nodes, especially sensor or actuator nodes, are sensitive to the energy consumption in the sense that a small decrease of working power might prolong the system lifespan significantly. Generally, the energy consumption of a sensor or actuator can be divided into three domains: sensing or actuating, wireless communication, and data processing. With the advancements in Micro-Electro-Mechanical Systems (MEMS) technology and integrated circuits, the power consumption of microprocessors or microcontrollers has reduced significantly. However, the energy consumption from wireless communication, especially RF front ends and antennas, is relatively harder to reduce, leading to the wireless module becoming a significant component for WBAN nodes. In addition, a WBAN system with a more efficient transmission power control scheme would be able to reduce the actual average transmitted power to have the same performance as in the conventional system (equivalently, it has a better performance with the same average transmitted power). Therefore, designing energy-efficient transmission schemes, especially Transmission Power Control (TPC) schemes, is important for reducing energy usage.

TPC refers to an adaptive method which optimizes the transmission power (Tx power) based on the channel conditions or QoS requirements. In fact, as a classical research topic, TPC has been extensively explored in the context of cellular networks and Wireless Sensor Networks (WSN). However, due to the presence of the human body, the extremely low power requirement and the need for high flexibility of transmission protocol designs, simple adoption of the TPC methods designed for other networks is not appropriate. For example, many existing TPC methods require the transmitting node to stay in active state to track the real-time channel condition to optimize the Tx power. However, in WBANs, the transmitter usually belongs to the sensor or actuator node, which is expected to be in sleep mode most of the time to save energy. Besides, to avoid the negative impact of electromagnetic radiation on the human body (e.g., temperature rise [6]), the Tx power should be restricted to comply with the limitation of Specific Absorption Rate (SAR) of local regulatory bodies (the limitation of U.S. Federal Communications Commission is 1.6 W/kg).

Among the TPC methods proposed for WBANs, adjusting the Tx power based on the channel condition to achieve a better trade-off between energy consumption and transmission reliability is considered as one of the major categories. The work presented in [7] is considered as the first TPC method designed for WBAN systems. In [7], Xiao firstly investigated the potential benefits of adaptive TPC for energy saving in body-wearable sensor devices used for medical monitoring, and then proposed a practical TPC method that adapts the Tx power according to the feedback Received Signal Strength Indicator (RSSI) values obtained from the sensor node. Specifically, the hub jointly considers the latest feedback RSSI value and previous average RSSI to estimate the new average RSSI (denoted as $\bar{R}$), and the new transmission level is configured based on the comparison between the new average RSSI and two pre-defined thresholds $T_{H}$ and $T_{L}$. If $\bar{R}$ drops below the lower threshold $T_{L}$, then the transmission power is doubled. In contrast, if $\bar{R}$ is above the upper threshold $T_{H}$, then the transmission power is reduced by a small fixed constant. Similarly, the TPC schemes proposed in [8,9,10,11,12,13,14] also utilize the comparison between historical RSSI record(s) and RSSI threshold(s) to reduce the power consumption while maintaining a high packet delivery ratio. In [15], the autocorrelation characteristics of on-body channels are taken into account to facilitate the TPC. The hub utilizes a correlation model to predict the future RSSI value, and then the transmission power level is adjusted based on the predicted value. Instead of using RSSI as the only indicator to estimate channel quality, Kim et al. [16] explored the combination of RSSI and LQI (Link Quality Indication), which may have a better performance in an environment with interference. Zou et al. [17], Yong et al. [18], Wang et al. [19] and Zhao et al. [20] explored mitigating the wireless interference by TPC in WBAN systems. Game theory is widely used for this kind of TPC methods. In these game theory based TPC schemes, the power control problem is modeled as a non-cooperative game, in which the cost function is designed based on both QoS requirement and energy constraint. Another group of TPC methods is adjusting the Tx power based on the change of human postures or motions [21,22,23,24,25]. This category of TPC methods is novel because the presence and movement of the human body are taken into account. However, in the dynamic scenarios, most of these works only consider the walking case which exhibits significant periodicity. The effectiveness in other complicated dynamic scenarios is not assessed. Besides, these works presume an effective activity recognition algorithm has been deployed, which also affects the feasibility. Besides, some studies try to use artificial neural network technology to optimize the TPC method in WBANs [26,27]. By training with massive RSSI data measured in human subjects, the channel quality estimation model is built to assist the adjust of Tx power.

Although applying a TPC approach at the sensor side can reduce energy consumption, TPC alone may not be sufficient to meet the stringent transmission reliability requirement of some WBAN applications, e.g., m-medical or e-health WBAN systems. In recent years, there are some works exploring relay-aided TPC methods in WBANs to achieve a better balance between energy consumption and transmission reliability. Zhang and Zhang [28] proposed a dynamic cooperative transmission scheme based on the last round channel condition. When the direct channel is too bad to complete the packet transmission, relay-aided two-hop transmission is introduced to improve the transmission reliability. In the scheme proposed in [29], the diversity gain from the two-hop relay link is utilized to decrease the outage probability, and radio Tx power control is integrated into sensor and relay nodes to extend the system lifetime and mitigate the signal interference from other WBAN systems. To achieve a better balance between transmission reliability and energy efficiency, the TPC scheme proposed in [30] jointly considers TPC, relay selection, and transmission time, and then converts the problem of controlling these parameters to a non-convex mixed-integer optimization problem.

Besides, integrating Network Coding (NC) and cooperative communication in the context of WBANs is an emerging research topic. One category of these works only considers a two-hop star topology for data streaming from sensor or actuator nodes to the hub [31,32,33,34,35]. Upon receiving the data packets from sensor or actuator nodes, the dedicated relay nodes perform eXclusive-OR (XOR) coding to these packets and transmit redundant packets to the hub. However, unlike massive WSNs, one-hop transmission is considered as an important component for WBAN systems. Moreover, these NC schemes are not adaptive to the channel condition, which impedes its potential to improve the transmission reliability in the WBANs with complex wireless channels variations. Another category of applying NC into WBAN system focuses on the cluster-based network architecture, in which the intermediate relay nodes are divided into multiple cooperative clusters to facilitate the cooperative communication [36,37,38,39,40]. Instead of forwarding original packets between consecutive clusters, the NC packets are utilized to reduce the packet retransmission time in the cluster-based schemes. However, this category of NC schemes appears to be simple extensions of the work proposed in [41] and the peculiarity of WBAN systems is not fully considered. First, as stated in [35], it is very difficult to make a clustering algorithm adaptive to the dynamic channel condition. Moreover, the network scale of WBAN is relatively smaller than other WSNs. Maximal two-hop transmission might be sufficient for most on-body channel transmissions. Thus, the feasibility of cluster-based schemes is questionable in the context of WBAN systems.

In this paper, we focus on the adaptive transmission protocol in real daily life WBAN scenarios. Inspired by the autocorrelation analyses presented in the literature [4,42,43], we propose an Autocorrelation-based Adaptive Transmission (AAT) scheme that jointly considers TPC, Dynamic Slot Scheduling (DSS) and two-hop cooperation. Firstly, the slot scheduling results are taken into account to optimize the transmission power level on the sensor side. Then, to further improve the transmission reliability, AAT provides an advanced cooperation option in which the two-hop transmission mechanism is designed to utilize spatial diversity. The newly proposed scheme is designed on the basis of IEEE 802.15.6. The channel datasets collected from real WBAN daily scenarios are imported into our simulation model to carry out the performance evaluation. The simulation results demonstrate that the AAT significantly improves the transmission reliability and enhances the energy efficiency. Besides, considering extensive research works which explore the application of NC technology in WBAN systems, we also provide a primary performance evaluation of the effectiveness and feasibility of NC technology in practical WBAN systems where both one-hop and two-hop transmissions are taken into account.

The main contributions of this paper are summarized as follows.

1. Motivated by the significant autocorrelation feature of on-body channels, we propose an adaptive TPC algorithm. The new TPC method optimizes the transmission power level based on the autocorrelation characteristic between two consecutive superframes.
2. Since DSS operations are carried out at the hub, a DSS mechanism is incorporated into the newly proposed TPC method to further optimize the transmission power.
3. The combination of TPC and two-hop relay cooperation is explored. We detail the protocol implementation on the basis of IEEE 802.15.6, including relay node selection, relayed node selection and adaptive allocation of relay slots (these terms are explained below).
4. The performance of the proposed protocol was evaluated using the measured channel data collected from the real daily scenarios. The evaluation results show that the newly proposed protocol achieves remarkably lower PLR (Packet Loss Ratio) while the energy consumption remains at a low level.

The rest of the paper is organized as follows. In Section 2, the system model is presented. Section 3 details the newly proposed AAT method. The performance evaluation results are presented in Section 4. Finally, Section 5 summarizes this paper.

## 2. System Model

In this section, the network architecture, channel model, and the energy consumption model are described.

### 2.1. Network Model

**Figure 1:**
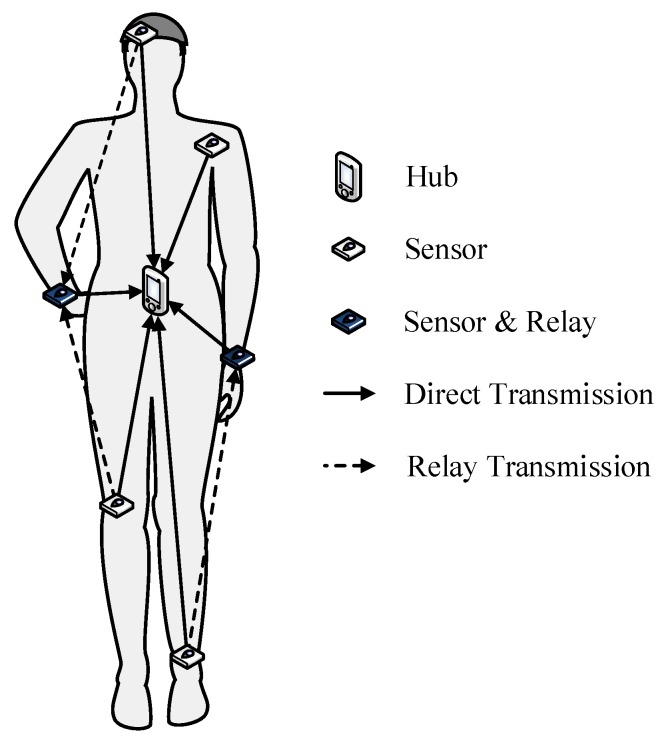
Deployment of wireless nodes.

In this paper, we consider the WBAN composed of *n* on-body sensor nodes (denoted as $SN_{1},SN_{2},\cdots ,SN_{n}$) and one hub node. All sensor nodes periodically upload monitoring data to the hub. Then, these monitoring data are transmitted to a data server or remote sink node by the hub. The off-body link is out of the scope of this paper. As shown in Figure 1, both one-hop (direct) transmission and two-hop (relay) transmission are considered in the star topology. In the case of Figure 1, the two sensors on the wrists act as the relay nodes. Specifically, the right wrist node is scheduled to relay the packets from the sensor located on the head and the sensor bound on right knee, while the left wrist node is assigned to relay the data packets from the left ankle. Note that a sensor node is called a relayed node only when its packets are scheduled to be relayed by a relay node. In this paper, the relay nodes are not dedicated; they are selected from sensor nodes.

The uplink transmissions for all sensor nodes are regulated in a TDMA fashion, where each sensor node has its own SUI (Scheduled Upload Interval) to transmit data to the hub, and the selected relay node has its SRI (Scheduled Relay Interval) to transmit the packets. As for the exchange of management and control packets, the CSMA/CA access method is performed before the SUIs of sensor nodes. Specifically, we adopt the beacon mode with superframes in the IEEE 802.15.6 standard [1], as this mode provides the most flexible options in terms of access phases. In the beacon mode with superframes, the timeline are divided by successive superframe (or beacon period), and each superframe begins with one beacon packet which is broadcast to all sensors by the hub node. Based on the standard of IEEE 802.15.6, the beacon packet is used to facilitate the time synchronization between the hub and sensor nodes. In this paper, we assume the time synchronization mechanism works well and time shift does not affect packet transmission between nodes. Moreover, in our newly proposed scheme, the beacon packet is also used to perform the the slot scheduling, relay/relayed nodes selection, and transmission power control, by embedding the configuration information into the beacon packet.

**Figure 2:**
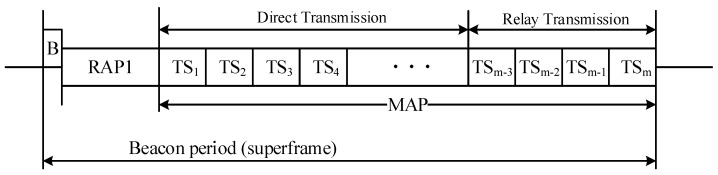
Superframe structure for AAT.

As shown in Figure 2, one active beacon period (superframe) consists of two access phases: Random Access Phase (RAP1) and Managed Access Phase (MAP). The CSMA/CA access method is adopted in the RAP1 phase, which occupies a fixed length after the transmission of beacon packet. The time period in the MAP phase is divided into two sub-phases: Direct Transmission Phase (DTP) and Relay Transmission Phase (RTP) to support both one-hop and two-hop transmissions. The SUIs assigned to sensor nodes are located in DTP. As demonstrated in [42], if we do not constrain the SUIs of all sensors to be at the same length, an unpredictable and significant data rate discrepancy between different sensors will exist. Therefore, in this paper, we consider a “fairness constraint”, in which all sensor nodes are allocated with SUIs of the same length. As for the RTP, the SRI assigned to the relay node is located in this phase to forward the packets from relayed nodes. If a sensor node also acts as a relay node, it would stay at the receiving (Rx) state during the SUI(s) of the relayed node(s) to listen and receive the packets from the relayed node(s), and it would try to send the received packets to the hub in RTP. On the other hand, if a sensor node does not act as a relay node, except for its own SUI period, the sensor would stay in sleep mode. Both DTP and RTP consist of multiple time slots with the same length. In the example in Figure 2, the last four time slots in the MAP are set as the RTP. If each sensor node is assigned with two time slots, the relay node is capable of forwarding the data packets from at most two relayed nodes during the RTP. To focus on evaluating the performance of TPC and relay cooperation, the No Acknowledgement (N-Ack) policy is adopted in the uplink. This means the packets from sensor nodes do not require an acknowledgement from the recipient, either immediately or later. Moreover, all hub, relay and sensor nodes are considered to operate in the half-duplex mode.

### 2.2. Channel Model

It is well-known that the Packet Loss Ratio (PLR) in WBAN system is affected by many factors, including environment factors (e.g., distance, human activity, body posture, and ambient objects) and technical factors (e.g., transmission power, error correction and coding, receiver sensitivity, and antenna gain). However, as the focus of this paper is on the influence of WBAN channel, we employ a ceteris paribus assumption with respect to other technical factors that do not affect the channel. Besides, it is not realistic to consider all environment factors when we want to evaluate the quality of wireless channels. Hence, similar to Xiao et al. [7], we choose Received Signal Strength Indicator (RSSI) as the indicator of the channel quality, and PLR is considered as a function of RSSI. Accordingly, PLR could remain around zero when RSSI is larger than a certain threshold. In this paper, this threshold is referred to as Rx sensitivity and we assume that a packet is correctly received when its RSSI value is greater than the Rx sensitivity. The RSSI of a packet can be expressed as follows:

(1) $$RSSI=P_{Tx}-Pathloss$$

where $P_{Tx}$ represents the transmission power of this packet at the transmitter and Pathloss is the path loss that the packet undergoes. Since the $P_{Tx}$ of a sensor node can be adjusted based on a TPC algorithm, the key part of modeling the on-body channel is describing the variation of path loss. As discussed before, the path loss of on-body channels is affected by many factors, such as the shadowing effect of human tissues and the mobility of the human body. In this work, the on-body channels in the daily scenarios are classified into two categories: direct channel and relay channel (cf. Figure 1). Specifically, the channel between the sensor and the hub is called the direct channel, while the channel from the sensor node to the relay node is denoted as the relay channel. Since the relay node is a sensor node, the channel between the relay node and the hub is also a direct channel.

#### 2.2.1. Direct Channel Model

**Figure 3:**
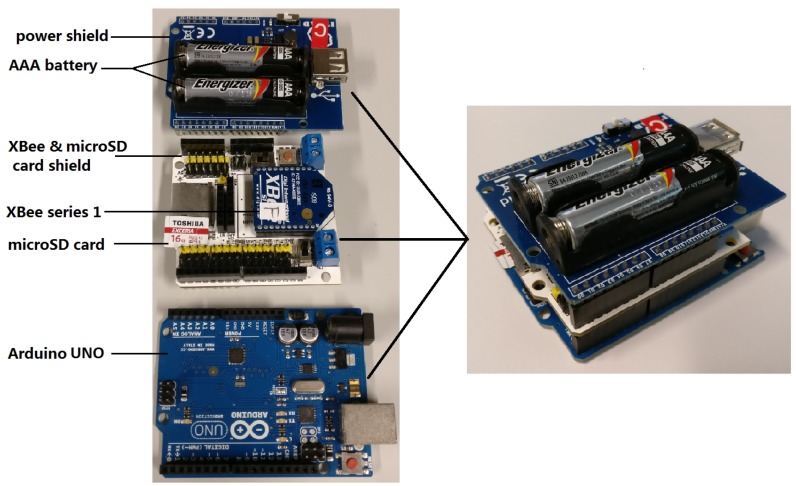
Components of the portable transceiver.

Due to high variability of on-body channels, neither distance-based nor other formula-based methods seems to be sufficient to describe the on-body channel condition, especially in the dynamic scenarios. Therefore, adopting channel datasets collected from the real daily scenarios to model the on-body channel is a better choice. The portable wireless transceivers introduced in [44] are used to collect the on-body channel gain data. The wireless transceivers work at the 2.4 GHz ISM band, which is one of the candidate carrier frequencies for the IEEE 802.15.6 BAN standard [1]. Moreover, compared to UWBs that use higher bands, the resolvable multipath and the Inter-Symbol Interference (ISI) can be neglected in the ISM 2.4 GHz frequency band [45,46]. The structure of the portable transceiver is shown in Figure 3. The main function of these transceivers is to transmit and receive continuous data packets to/from each other and record the RSSI values into a Micro SD card. More detailed description of the hardware can be found in [42,44,47].

**Figure 4:**
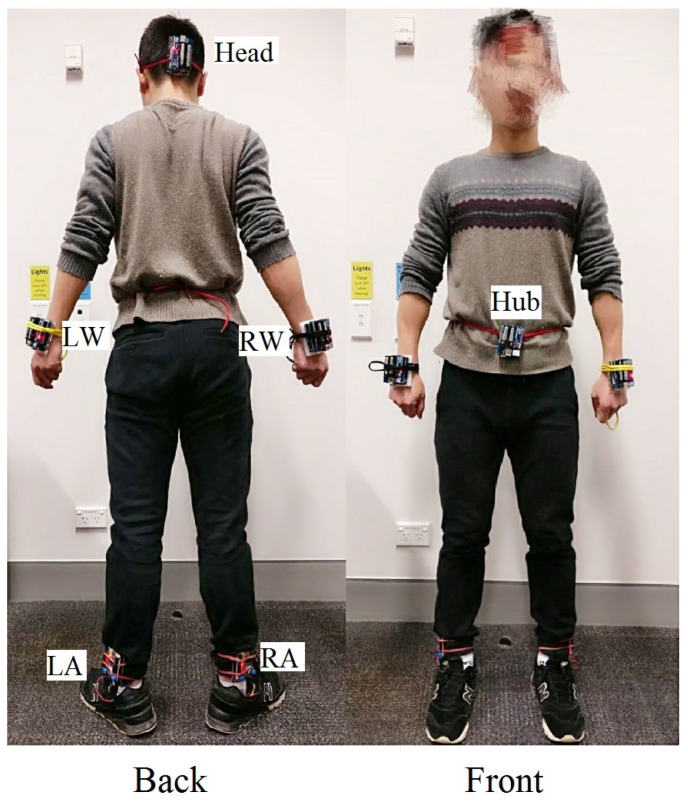
The measurement for direct channels.

Corresponding to Figure 1, the deployment of the transceivers is depicted in Figure 4, where the transmitter (acting as the hub) is placed on the abdomen and five receivers (acting as sensor nodes) are mounted on limbs and head. When the measurement begins, the transmitter continuously broadcasts sample packets to the five receivers with the transmission (Tx) power of 0 dBm, and the sample packets transmission frequency is 200 Hz (i.e., sending 200 packets per second). Upon receiving the sample packets, the receivers record their packet sequence numbers, timestamps, and RSSI values into a text trace file. For each measurement, there are five trace files, and we call the five trace files as a channel dataset. More details about the measurements and the channel datasets can be found in [4]. In this study, 16 individual channel datasets collected from real daily WBAN scenarios are considered. Moreover, as the on-body channels show a prominent reciprocity in narrowband communication environments [48,49], the channel profiles of downlink and uplink are approximately the same. Therefore, these channel datasets are utilized to model the on-body channels, including downlink and uplink.

#### 2.2.2. Relay Channel Model

Our portable wireless transceivers work in a half-duplex mode, and the path losses of all direct channels are recorded by broadcasting sample packets from the hub to the sensors. The broadcasting method has the advantage that the path loss data for different links are synchronous, but it also means the path loss information between different sensors themselves is missing in the channel datasets. Accordingly, the relay channels are not represented in these datasets. In this paper, we mainly focus on the one-hop channel, i.e., the channel between the sensor and the hub, and the channel from one sensor to another sensor is considered only when the second sensor is chosen as the relay node. Therefore, we adopt a relatively straightforward method to represent the channel condition from the relayed node to the relay node. One parameter named $PLR_{r}$ is used to represent the packet loss ratio for this channel between a relayed node and a relay node. Moreover, as we focus on the channel condition from source or relay nodes to the hub, the parameters $PLR_{r}$ for all relay channels are assumed to be the same. As a result, the success of one two-hop transmission is decided by not only the channel gain recorded in the channel datasets but also the parameter $PLR_{r}$.

### 2.3. Energy Consumption Model

There are a number of energy consumption models in the literature which can be applied to WBANs, e.g., the models used in [30,50,51,52,53,54,55,56]. However, most of these works do not take the energy consumption of the radio state transition and the energy consumption in sleep mode into account, and the transmission time is simply equal to the time interval allocated to the transmitter. To achieve a more accurate calculation of energy consumption, we adopt the energy consumption model in the network simulator Castalia [57] for WBANs. Besides, as the hub node is usually considered to be less constrained by energy, we focus on the energy consumption of sensor nodes.

**Figure 5:**
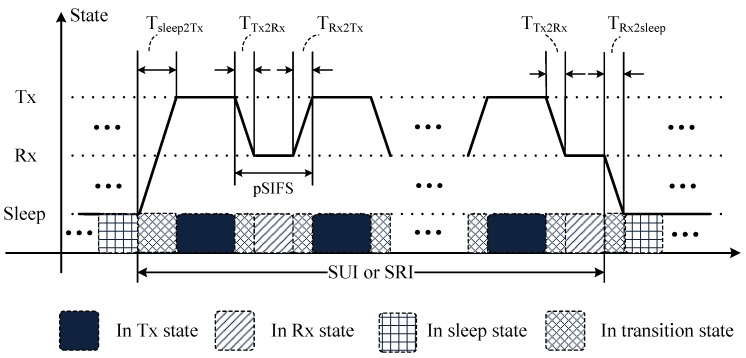
Energy consumption in one SUI or SRI.

Because the MAC layer works in the beacon mode with superframes and the superframe length is defined as a fixed parameter in IEEE 802.15.6, taking the energy consumption in one superframe is enough to explain the energy consumption model. In each superframe, the energy consumption for one sensor node consists of four parts: the energy consumption in the Tx state, the energy consumption in the Rx state, the energy consumption in the sleep state, and the energy consumption used to complete the state transition. Hence, the energy consumption in one superframe for one sensor can be expressed as follows

(2) $$E_{sum}=E_{Tx}+E_{Rx}+E_{sleep}+E_{transition}$$

where $E_{Tx}$, $E_{Rx}$, $E_{sleep}$ and $E_{transition}$ represent the four components of the whole energy consumption, respectively. Figure 5 illustrates the variation of radio states during one SUI or SRI. $T_{x2y}$ in the figure refers to the transition delay from State *x* to State *y*, and pSIFS is the time interval between two consecutive transmissions. As the default active state is Rx state, the radio returns to the Rx state after completing each transmission.

We first detail the calculation of $E_{Tx}$ in one superframe as follows

(3) $$E_{Tx}=P_{Tx}\times T_{Tx}$$

where $P_{Tx}$ stands for the transmission power that the sensor node adopts in the superframe and $T_{Tx}$ represent the time in the Tx state. Note that the value of $P_{Tx}$ may vary in different superframes since TPC is considered in this study. The length of $T_{Tx}$ varies with the role of the sensor in that beacon period. If the node does not act as a relay node, $T_{Tx}$ can be expressed as

(4) $$T_{Tx}=\frac{L_{p}}{R}\times \left\lfloor 1+\frac{SUI-(T_{sleep2Tx}+L_{p}/R)}{L_{p}/R+pSIFS}\right\rfloor$$

where *R* (Kbps) refers to the transmission rate, which is assumed to be the same for all sensors. $L_{p}$ (Kbits) denotes the length of one data packet or frame. $\frac{L_{p}}{R}$ is the time of transmitting one data packet. As the sensor only transmits packets during its SUI, the number of transmitting packets is $\left\lfloor 1+\frac{SUI-(T_{sleep2Tx}+L_{p}/R)}{L_{p}/R+pSIFS}\right\rfloor$ which is denoted as $PN_{1}$. Hence, the time duration in the Tx state is $\frac{L_{p}}{R}\times PN_{1}$. On the other hand, if the sensor is selected as a relay node, it not only transmits its own data packets during its SUI but also forwards the relaying packets. In this case, the $T_{Tx}$ can be calculated as

(5) $$T_{Tx}=\frac{L_{p}}{R}\times \left(PN_{1}+\left\lfloor 1+\frac{SRI-(T_{sleep2Tx}+L_{p}/R)}{L_{p}/R+pSIFS}\right\rfloor \right)$$

If we denote the number of relaying packets as $PN_{2}=\left\lfloor 1+\frac{SRI-(T_{sleep2Tx}+L_{p}/R)}{L_{p}/R+pSIFS}\right\rfloor$, then $T_{Tx}$ of a relay node can be expressed as $\frac{L_{p}}{R}\times \left(PN_{1}+PN_{2}\right)$.

Next, we detail the calculation of $E_{Rx}$, which can be expressed by

(6) $$E_{Rx}=P_{Rx}\times T_{Rx}$$

where $P_{Rx}$ denotes the power when sensor node is in the Rx state, and $T_{Rx}$ represents the time duration in this state. Similar to the calculation of $T_{Tx}$, whether a node acts as a relay node affects the length of $T_{Rx}$. If the node does not act as a relay node, the $T_{Rx}$ is given by

(7) $$\begin{array}{cc} T_{Rx} & =\left(PN_{1}-1\right)\left(pSIFS-T_{Tx2Rx}-T_{Rx2Tx}\right)\\ & \;+\left(SUI-T_{sleep2Tx}-\frac{L_{p}}{R}-T_{Tx2Rx}-\left(PN_{1}-1\right)\times \left(pSIFS+\frac{L_{p}}{R}\right)\right) \end{array}$$

Partitioned by the plus sign, $T_{Rx}$ in Equation (7) has two parts. The first part is the Rx time between two consecutive packet transmissions, and the second part is the Rx time at the end of SUI, which is not long enough to transmit one data packet. The calculation of $T_{Rx}$ for a relay node is more complicated than a sensor node. It not only includes the Rx time during SUI and SRI but also includes the time spent on receiving the data packets from relayed nodes

(8) $$\begin{array}{cc} T_{Rx} & =\left(PN_{1}-1\right)\left(pSIFS-T_{Tx2Rx}-T_{Rx2Tx}\right)\\ & \;+\left(SUI-T_{sleep2Tx}-\frac{L_{p}}{R}-T_{Tx2Rx}-\left(PN_{1}-1\right)\times \left(pSIFS+\frac{L_{p}}{R}\right)\right)\\ & \;+\left(PN_{2}-1\right)\left(pSIFS-T_{Tx2Rx}-T_{Rx2Tx}\right)\\ & \;+\left(SRI-T_{sleep2Tx}-\frac{L_{p}}{R}-T_{Tx2Rx}-\left(PN_{2}-1\right)\times \left(pSIFS+\frac{L_{p}}{R}\right)\right)\\ & \;+(SRI-T_{sleep2Rx}\times \alpha ) \end{array}$$

Partitioned by the plus signs, the first two parts are similar to the Equation (7), which represent the Rx time during its SUI. The middle two parts are the Rx time during its SRI to forward the relaying packets to the hub. The last part is the duration when the relay node stays in the Rx state to receive the data packets from relayed node(s). Note that we introduce a parameter α, which varies with the number of relayed nodes and whether their SUIs are adjacent. For example, if two sensor nodes are selected as the relayed nodes and their SUIs are not adjacent, α is two. On the other hand, if their SUIs are adjacent, α is one.

Next, we calculate the energy consumption of the state transition. We first detail this kind of energy consumption for a sensor node, which can be expressed as follows

(9) $$\begin{array}{cc} E_{transition} & =P_{sleep2Tx}\times T_{sleep2Tx}\\ & \;+\left(PN_{1}-1\right)\left(P_{Tx2Rx}\times T_{Tx2Rx}+P_{Rx2Tx}\times T_{Rx2Tx}\right)\\ & \;+P_{Tx2Rx}\times T_{Tx2Rx}\\ & \;+P_{Rx2sleep}\times T_{Rx2sleep} \end{array}$$

Partitioned by the plus signs, the first and the last two parts in Equation (9) represent the energy consumption of state transitions before and after data packet transmissions, respectively. The second part is the energy consumption of the state transition during the transmissions of consecutive data packets. Next, if the node is selected as the relay node, the energy consumption used to complete the radio state transitions is calculated by:

(10) $$\begin{array}{cc} E_{transition} & =P_{sleep2Tx}\times T_{sleep2Tx}\\ & \;+\left(PN_{1}-1\right)\left(P_{Tx2Rx}\times T_{Tx2Rx}+P_{Rx2Tx}\times T_{Rx2Tx}\right)\\ & \;+P_{Tx2Rx}\times T_{Tx2Rx}\\ & \;+P_{Rx2sleep}\times T_{Rx2sleep}\\ & \;+P_{sleep2Tx}\times T_{sleep2Tx}\\ & \;+\left(PN_{2}-1\right)\left(P_{Tx2Rx}\times T_{Tx2Rx}+P_{Rx2Tx}\times T_{Rx2Tx}\right)\\ & \;+P_{Tx2Rx}\times T_{Tx2Rx}\\ & \;+P_{Rx2sleep}\times T_{Rx2sleep}\\ & \;+(P_{sleep2Rx}\times T_{sleep2Rx}+P_{Rx2sleep}\times T_{Rx2sleep2})\times \alpha \end{array}$$

The first eight lines of Equation (10) represent the energy consumptions of the state transitions during its SUI and SRI. The last line calculates the energy consumption of the state transition during the SUI(s) of the relayed node(s). The value of α varies with the number of relayed nodes and whether their SUIs are adjacent. Its meaning is explained by Equation (8).

Finally, the energy consumption in the sleep state can be expressed as

(11) $$E_{sleep}=P_{sleep}\times T_{sleep}$$

where $P_{sleep}$ represents the power when a sensor node is in the sleep mode, and $T_{sleep}$ is the time duration in the sleep mode. The $T_{sleep}$ in one superframe is not provided in this section, as it can easily be calculated by subtracting the time in other three radio states from the superframe length.

In this study, we paper the energy consumption parameters from the CC2420 radio chip [58]. The Tx power (when the module is in Tx state), current consumption, and corresponding working power in different states for CC2420 are listed in Table 1. As shown in Table 1, the current consumption of the module changes with the state of the module. The current consumption of sleep state is significantly lower than those of Rx state and Tx state, which also provides the rationale of reducing unnecessary active time, i.e., the time that the module is in Tx or Rx state. Specifically, when the module is in Tx state, decreasing the Tx power leads to the reduction of current consumption, which also results in the decrease of working power of the whole module. Besides, it is worth noting that the Tx power of −1 dBm, −3 dBm and −7 dBm are added by the network simulator Castalia [57]. These levels of Tx power do not exist in the real CC2420 module. We also provide the transition delay and power between different states in Table 2.

## 3. Proposed Transmission Scheme

Given that the hub is typically more powerful than the sensor nodes in terms of storage and computational resources, it is desirable to push more control and computational tasks to the hub. In the proposed AAT scheme, except for the ability of changing transmission power and optional relaying at the sensor side, the hub side implements all control and calculation operations, including channel condition prediction, transmission power level decision, adaptive relaying scheduling, etc. Specifically, AAT scheme consists of three main steps, which are summarized as follows:

1. The hub keeps track of the channel conditions from all sensor nodes, and then predicts the channel condition in the next TDMA round based on a temporal autocorrelation model.
2. Based on these predicted channel conditions, the hub adjusts the transmission power and reschedules the SUI order for all sensors.
3. After configuring the transmission power and SUI order, some channels may still be predicted to be in an outage state. In this case, the hub selects the relay and relayed nodes based on the predicted channel conditions, and then schedules the relay slots adaptively.

### 3.1. Channel Condition Prediction

The temporal autocorrelation model used to predict the channel condition is firstly introduced. Suppose the sensor node $SN_{i}$ transmits a data packet to the hub with the transmission power $P_{Tx}$, and the hub receives the packet with a signal power of $P_{Rx}$. Then, the channel gain for the channel “$SN_{i}$-Hub” is defined as

(12) $$G_{i}{|}_{dB}=P_{Rx}{{|}_{dBm}-P_{Tx}|}_{dBm}$$

Meanwhile, as proved in [48,59,60,61], the lognormal distribution provides a good fit for a long-term average on-body channel gain. Therefore, the channel gain (in dB) in the channel “$SN_{i}$-Hub” can be described by a Gaussian random variable (r.v.)

(13) $$G_{i}{|}_{dB}∼N\left(\mu_{i},\sigma_{i}^{2}\right)$$

where $\mu_{i}$ and $\sigma_{i}$ are the mean and standard deviation of the channel gain, respectively. Note that both $\mu_{i}$ and $\sigma_{i}$ depend directly on the type of human activity, the position of transmitting and receiving nodes, and propagation environment. As proved in [62], the variation of the channel gain can be considered as a Wide Sense Stationary (WSS) process within 500 ms. If the superframe length is less than 250 ms, the channel gain for this channel in the next superframe follows the same distribution

(14) $$\begin{array}{ccc} G_{i}\left(S\right) & ∼ & N(\mu_{i},\sigma_{i}^{2}) \end{array}$$

(15) $$\begin{array}{ccc} G_{i}\left(S+1\right) & ∼ & N(\mu_{i},\sigma_{i}^{2}) \end{array}$$

where $G_{i}\left(S\right)$ and $G_{i}\left(S+1\right)$ are the channel gains in the superframe *S* and S+1, respectively. Therefore, the joint distribution of the two channel gains recorded during two consecutive superframes can be expressed as

(16) $$\left(G_{i}\left(S\right),G_{i}\left(S+1\right)\right)∼N\left(\mu_{i},\mu_{i},\sigma_{i}^{2},\sigma_{i}^{2},\rho_{i}\right)$$

where $\rho_{i}$ denotes the autocorrelation coefficient between the two channel gains recorded in the two adjacent superframes. Furthermore, the conditional distribution of $G_{i}\left(S+1\right)$ can be deduced to

(17) $$\begin{array}{c} G_{i}\left(S+1\right){|}_{G_{i}\left(S\right)}∼N\left(\left(1-\rho_{i}\right)\mu_{i}+\rho_{i}G_{i}\left(S\right),\left(1-\rho_{i}^{2}\right)\sigma_{i}^{2}\right) \end{array}$$

Now, the expected value of the channel gain in the next superframe S+1 can be estimated by $\left(1-\rho_{i}\right)\mu_{i}+\rho_{i}G_{i}\left(S\right)$. In fact, Equation (17) can be considered as a “lite version” of the temporal autocorrelation model (TAM) used in [42]. Instead of adding an extra wake-up period at the end of each time slot to calculate the autocorrelation coefficient between different time slots, the TAM proposed in this paper only needs the regular wake-up period, which would reduce the energy consumption.

### 3.2. Transmission Power Control

To estimate the channel condition in the next superframe using Equation (17), the following parameters are required in the hub side: $G_{i}\left(S\right)$, $\mu_{i}$, $\sigma_{i}$ and $\rho_{i}$. Firstly, the latest channel gain recorded in the previous superframe is chosen as $G_{i}\left(S\right)$. Then, channel gain mean $\mu_{i}$ and standard deviation $\sigma_{i}$ can be estimated by the sample mean (${\hat{\mu }}_{i}$) and sample standard deviation (${\hat{\sigma }}_{i}$).

(18) $$\begin{array}{ccc} {\hat{\mu }}_{i} & = & \bar{G_{i}}=\frac{1}{N}\sum_{x=1}^{N}G_{i}\left(x\right) \end{array}$$

(19) $$\begin{array}{ccc} {\hat{\sigma }}_{i} & = & \sqrt{\frac{1}{N}\sum_{x=1}^{N}{\left(G_{i}\left(x\right)-\bar{G_{i}}\right)}^{2}} \end{array}$$

where $G_{i}\left(x\right)\;\left(x=1,2,\cdots ,N\right)$ are the historical channel gain records, and *N* is the sample size. In fact, there exists a trade-off between accuracy and timeliness when choosing the sample size. On the one hand, with the increase of sample size, more data points are utilized to calculate the channel autocorrelations, which may appear to result in a more accurate estimation of autocorrelation. On the other hand, since the sampling frequency is fixed, a bigger sample size means these data points are collected from a wider time span. However, as demonstrated in [62], on-body channels show a low probability of WSS assumption outside the time span of 500 ms. In other words, the timeliness of autocorrelation calculation is weakened with the increase of sample size. In this paper, we adopt the optimal sample size used in [42], i.e., recording the channel gain values of the past 2 s. For example, if the superframe length is 80 ms, the hub stores the latest ⌊2000/80⌋=25 channel gain values. Next, we calculate the autocorrelation coefficient $\rho_{i}$ based on the following equation

(20) $$\rho_{i}=\frac{\sum_{x=1}^{N-1}\left(G\left(x\right)-{\hat{\mu }}_{i}\right)\left(G\left(x+1\right)-{\hat{\mu }}_{i}\right)}{\sum_{x=1}^{N}{\left(G\left(x\right)-{\hat{\mu }}_{i}\right)}^{2}}$$

where $G_{i}\left(1\right)\cdots G_{i}\left(N\right)$) are sample channel gain values recorded in *N* consecutive superframes.

At the beginning of each superframe, the hub performs the calculations of $G_{i}\left(S\right)$, ${\hat{\mu }}_{i}$, ${\hat{\sigma }}_{i}$ and $\rho_{i}$ for each channel, i.e., $SN_{i}-hub,\;i=\left(1,2,\cdots ,n\right)$. Then, the adaptive algorithm (Algorithm 1) is carried out to decide the transmission power level for each sensor in the current superframe.

**Algorithm 1:** Adaptive transmission power control method.

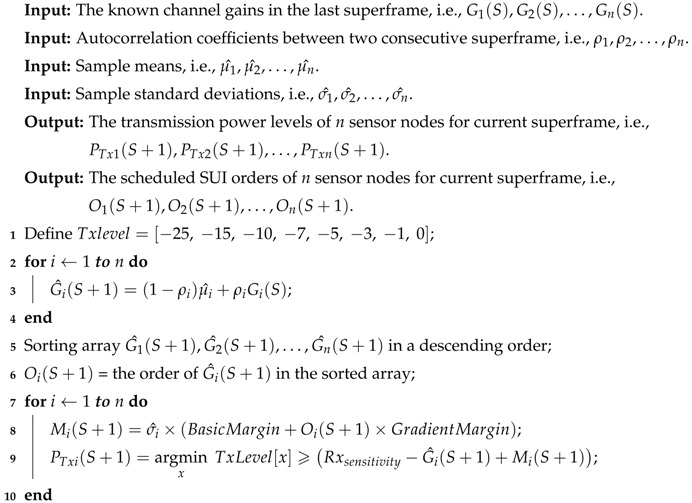



Algorithm 1 requires the following four inputs: the last known channel gain, autocorrelation coefficients between two consecutive superframes, the sample means, and the sample standard deviations. These inputs are used to schedule the SUI order and adapt the transmission power. Correspondingly, there are two output arrays: transmission power array ($P_{Tx1}\left(S+1\right),P_{Tx2}\left(S+1\right),\cdots ,P_{Txn}\left(S+1\right)$) and SUI order array ($O_{1}\left(S+1\right),O_{2}\left(S+1\right),\cdots ,O_{n}\left(S+1\right)$). Specifically, the radio output powers in different transmission levels are predefined on Line 1. Since the CC2420 chip is adopted, the TxLevel array is configured based on the parameters listed in Table 1. Lines 2–4 compute the expected value of channel gains in the current superframe by adding the sample mean to the last known channel gain, and the weights of these two parts are adjusted by the value of autocorrelation coefficient. Lines 5–6 readjust the order of sensors’ SUIs so that the sensor with the best channel quality, i.e., the highest expectation of channel gain, is scheduled in the first position of DTP (Direct Transmission Phase), and the sensor with the worst channel quality is scheduled at the end of DTP. The rationale behind such scheduling is that all “bad” links are given a longer time to recover (i.e., to get out of the outage), while the “good” links are assigned to front positions to ensure a high probability of successful delivery. Lines 7–10 are used to finalize the transmission power levels for the *n* sensor nodes. $M_{i}\left(S+1\right)$ refers to the transmission power margin for $SN_{i}$, which takes the channel variation into account to ensure that the RSSI is higher than the Rx sensitivity (RSSI threshold). BasicMargin and GradientMargin are two margin parameters used to adjust the sensitivity of TPC to the order of SUI. In this paper, the two margin parameters are configured at fixed values. Finally, the minimum predefined output power levels that are equal or higher than the target transmission powers, i.e., ${\hat{G}}_{i}\left(S+1\right)+M_{i}\left(S+1\right),\;i=1,2,\cdots ,n$, are selected. In addition, as we adopt “Bubble Sorting” algorithm on Line 5, the worst-case time complexity of this algorithm is $O(n^{2})$, where *n* is the number of sensors.

After performing Algorithm 1 at the beginning of each beacon period, the hub obtains the two arrays: $\left[P_{Tx1}\left(S+1\right),P_{Tx2}\left(S+1\right),\cdots ,P_{Txn}\left(S+1\right)\right]$ and $\left[O_{1}\left(S+1\right),O_{2}\left(S+1\right),\cdots ,O_{n}\left(S+1\right)\right]$. These two arrays are embedded into the beacon packet and broadcast to all sensor nodes. Upon receiving the beacon packet, sensor nodes set their radio state timer, control data packet uploading based on their SUI scheduling, and their transmission power level based on the configured values.

### 3.3. Two-Hop Cooperative Option for AAT

The major advantage of TPC methods lies in significantly reducing the energy consumption of sensor nodes. However, WBAN applications also have a stringent requirement in terms of PLR. As shown in Section 4, when the channel is in deep fading, the PLR might still be over 10% even with the maximum transmission power. In this case, considering TPC by itself may not be enough to achieve high transmission reliability. As a classical mechanism to decrease the PLR, retransmission schemes can be adopted after detecting the packet loss. However, retransmissions are only effective when the outage duration is significantly shorter than the packet delivery deadline, which may not be valid in the daily WBAN scenarios. As shown in [42], over 50% of outage durations exceed 10 ms in these scenarios. In this case, the immediate retransmission may have a high probability of failure, which results in energy wastage. On the other hand, the delayed retransmission may cause severe transmission delay, which is also unacceptable for many WBAN applications. Accordingly, cooperative transmission approaches are presented as a promising solution to further improve the transmission reliability. As cooperative communication has the advantage of spatial diversity, the packets from nodes in an outage duration could be delivered by the relay node instead.

In AAT schemes, the two-hop cooperative communication is considered as an option when a lower PLR is required or a severe channel fading is detected. As a relay node not only sends its own packets but also assists the relayed nodes, the extra energy consumption resulting from acting as a relay node should be carefully considered. In particular, two aspects should be designed when cooperative communication is jointly considered with the TPC method. First, the relay node must remain awake to receive the packets from the relayed node, but, when the direct channel from the relayed node to the hub is in a good condition, this extra awake period is a waste of energy. Therefore, choosing the relayed node(s) effectively could reduce unnecessary energy consumption. Second, relay node selection is of great importance in terms of the system lifetime. If the relay node is selected simply based on the channel condition or the distance to the hub node, some sensor nodes may run out of energy much more quickly than the other nodes due to their heavy traffic load and the whole system lifetime is shortened. Considering the aforementioned two aspects, the following two-hop cooperative mechanism is proposed.

1. **Relayed node selection:** When the estimated channel gain in the next superframe (${\hat{G}}_{i}\left(S+1\right)$) is below the Rx sensitivity, the corresponding sensor node is selected as a relayed node. The rationale for the selection is that, when ${\hat{G}}_{i}\left(S+1\right)$ is smaller than the Rx sensitivity, even if the transmission power is set to the maximum (0 dBm), the $RSSI=0+{\hat{G}}_{i}\left(S+1\right)$) would still be below the Rx sensitivity. Note that the maximal number of the relayed nodes is limited by the length of DTP. If DTP is *q* times longer than one SUI, then at most *q* sensor nodes can be set as relayed node. Therefore, if more than *q* sensors satisfy the above condition for relayed node selection, the *q* sensors with the worst channel gains are selected.
2. **Relay node selection:** In this paper, only one relay node is considered. Besides, to avoid some sensor nodes to be selected as relay nodes frequently, all sensor nodes whose predicted channel gains are greater than the Rx sensitivity are the candidate for becoming a relay node. Then, the actual relay node is randomly selected from these candidates.
3. **Time slot scheduling for the relay node:** There is no additional action for a sensor node when the node is selected as a relayed node. However, there are some extra radio state transitions and packet relaying tasks for the selected relay node. Similar to the TPC decision broadcast, the relayed and relay nodes selection results are also included in the beacon packet. Upon receiving the beacon packet, all sensor nodes check whether they are selected as a relay node. If yes, the relay node would turn to the Rx state during the SUI(s) of the relayed node(s), and then relay the received packet(s) to the hub during RTP.

In short, at the start of each beacon period, the hub estimates the channel gains for each link based on the temporal autocorrelation model and performs Algorithm 1 to optimize the transmission power and SUI scheduling for the next superframe. Moreover, if the two-hop cooperative option is selected, the relay and relayed nodes are selected to further improve the transmission reliability. Then, all these configurations are inserted into the beacon packet, which is broadcast to all sensor nodes. Finally, the sensor nodes adapt the transmission parameters based on the configuration information in the beacon packet.

## 4. Performance Evaluation

In this section, the performance of AAT is evaluated through simulations and compared with other transmission methods. To improve the authenticity of performance evaluation, the 16 channel datasets ($CD_{1}$–$CD_{16}$) collected from our measurement campaigns were imported into the simulation model to represent the variation of actual on-body channels in the daily scenarios. More details about the measurement campaigns can be found in [4,44]. We compared the performance of the following methods with or without two-hop cooperation:

1. **Static scheme:** The hub does not control the transmission power level of the sensor nodes. The transmission power is fixed at a predefined value, which is selected to be at 0 dBm. Besides, the static scheme does not change the slot scheduling of sensors. In other words, the permutation of SUIs is fixed after assigned randomly in the first superframe.
2. **Xiao’s scheme:** This TPC method, as proposed in [7], adapts the transmission power level based on the variation of channel conditions. Specifically, the hub node alters the transmission power level according to the feedback RSSI value obtained from the sensor node. The latest feedback RSSI value and previous average RSSI are jointly considered to estimate the new average RSSI ($\bar{R}$), and the new transmission power level is configured based on the comparison between the new average RSSI and the two configured thresholds $T_{H}$ and $T_{L}$. If $\bar{R}$ drops below the lower threshold $T_{L}$, then the transmission power is doubled. If $\bar{R}$ is above the upper threshold $T_{H}$, the transmission power is reduced by a fixed constant. Based on different weighting configurations (i.e., $\alpha_{u}$ and $\alpha_{d}$) between the latest RSSI and previous $\bar{R}$, three TPC schemes, namely *Conservative*, *Balance* and *Aggressive*, are designed for different applications with different requirements in terms of PLR and energy consumption. Considering the significant autocorrelation of on-body channels, the *Balance* scheme with $\alpha_{u}=0.8$ and $\alpha_{d}=0.8$ was chosen as the TPC method for comparison.
3. **AAT scheme:** This is the adaptive transmission scheme proposed in this paper. Unlike Xiao’s scheme, a temporal autocorrelation model is adopted to adjust the weight between the latest channel gain and the sample mean of historical channel gains. Moreover, the permutation of SUIs is also taken into account to optimize the transmission power margin. The two margin parameters, i.e., BasicMargin and GradientMargin, were set to 0.6 and 0.2, respectively.
4. **Ideal scheme:** In the ideal scheme, the hub knows all the channel gain information for the next superframe. Accordingly, the transmission power could be set at the most suitable level, which not only avoids unnecessary energy wastage but also keeps the PLR at the lowest level. This scheme was considered to explore the upper bound of TPC approaches. Note that this method is infeasible for real WBAN systems because it assumes perfect prediction of future states.

### 4.1. Simulation Model and Configurations

To evaluate the performance of the newly proposed adaptive transmission scheme, a comprehensive simulation model from wireless channels to the application layer was built based on the Castalia framework [57]. All the important default parameters of the protocol stack and hardware are listed in Table 3. On the wireless channel layer, the “TraceChannel” model was selected, in which the on-body channels are simulated by the channel dataset collected from our measurement campaign. As one channel dataset contains the channel gain variation in a time period of 3600 s (1 h), the simulation time was also set to 3600 s. The functions of application and routing layers are relatively simple. The application layer generates sample data packets with the rate of “PacketRate”, and the routing layer forwards packets between application layer and MAC layer. In the MAC layer, the superframe length was set to 80 ms, and the length of one time slot was 5 ms. Hence, one superframe consisted of 16 time slots, in which the first two time slots were set as the RAP1 period, the middle ten time slots belonged to the DTP and the last four time slots were RTP. The 10 time slots in DTP were assigned to five sensor nodes, and the SUI for one sensor node was two time slots, i.e., 10 ms. Note that the length of the active time slot in RTP depends on how many sensor nodes are estimated to be in outage for the current superframe. If one sensor node were predicted to be in outage, only two slots in the RTP period would be activated for the transmission from the relay node to the hub. If more than one sensor node were predicted to be in outage, then all four time slots would be activated. As for the radio layer, all parameters were set based on a low power RF transceiver module: CC2420 [58]. Taking the overheads of different layers into account, the size of one data frame was 128 bytes (105 + 10 + 7 + 6), thus the transmission delay for one frame was (128×8)/250=4.096 ms. Taking the guard time and pSIFS into account, the sensor nodes only transmit one data frame in one time slot. Consequently, the sensor node transmits two data frames to the hub during its SUI.

### 4.2. Simulation Results

Three performance evaluation metrics were considered: PLR (Packet Loss Ratio), energy consumption and energy efficiency. Specifically, the PLR presents the average PLR value of the five sensor nodes and energy consumption refers to the sum energy consumed by the five sensors. Energy efficiency (in KB/J) is defined as the ratio of data received in the hub side and the total power consumed by the five sensors. In our simulation model, the Rx sensitivity was configured as a variable value (not a fixed value) to evaluate the performance of AAT in a range of Rx sensitivity values.

#### 4.2.1. Adaptive Transmission without Cooperation

**Figure 6:**
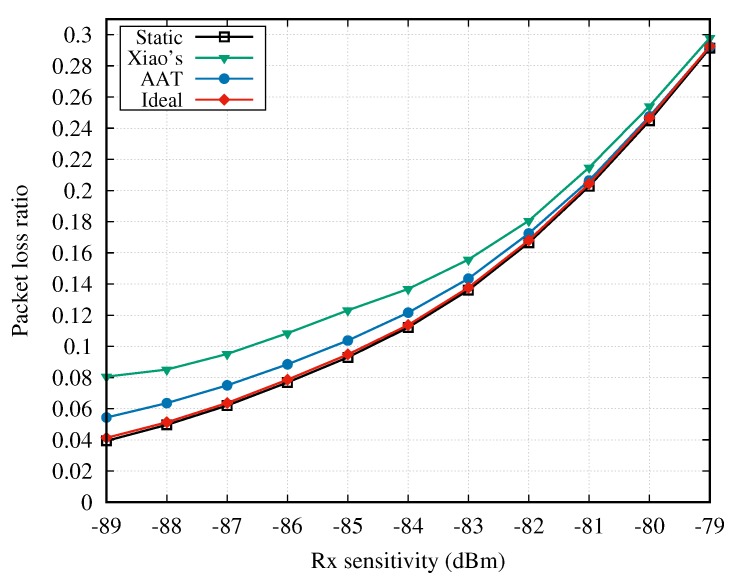
Packet loss ratio vs. Rx sensitivity.

We first considered the situations that the two-hop cooperative transmission is not used. The newly proposed AAT scheme was compared with the static or other TPC schemes by importing an example channel dataset, i.e., $CD_{1}$, into the simulation model. Figure 6 shows the average PLR of the five sensor nodes when the Rx sensitivity of hub varies. It can be seen that the PLR rises quickly with the increase of the Rx sensitivity. The ideal and static schemes achieve almost the same PLR performance, which is also the lowest among the four schemes. The lowest PLR value achieved by the ideal scheme is the upper bound for TPC approaches. The static method with the transmission power of 0 dBm achieves the best PLR performance, but at the price of a significant waste of energy, which is illustrated below. The PLR performance of the proposed AAT is close to the ideal method and much lower than Xiao’s scheme since AAT uses channel autocorrelation to adaptively adjust the transmission power and the SUI scheduling, as explained before. Another observation is that the PLR gap between AAT and Xiao’s scheme becomes smaller with the increasing value of Rx sensitivity. In other words, the performance advantage of the AAT scheme is more significant when Rx sensitivity is at a low level. For example, when the Rx sensitivity is −89 dBm, the PLR of AAT is 0.05434 while that of Xiao’s scheme is 0.08058, which is a 32.56% improvement in terms of PLR.

**Figure 7:**
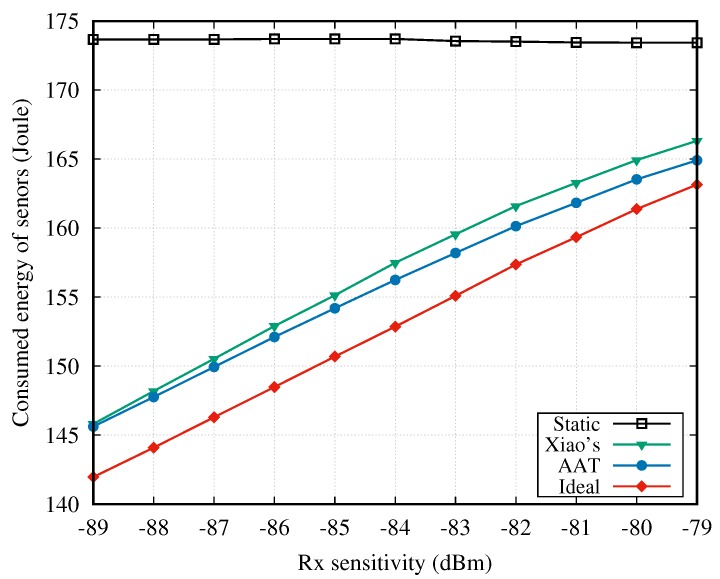
Energy consumption vs. Rx sensitivity.

Figure 7 shows the energy consumption of five sensors as a function of Rx sensitivity, when $CD_{1}$ is imported to the simulation model. It can be seen in the figure that the energy consumption of all TPC methods rise with the increase of Rx sensitivity, but always below that of the static method. Combining Figure 6 and Figure 7, the static method achieves the best PLR performance at a heavy price of energy consumption. The ideal method consumes the least energy as it always sets the Tx power at the optimal level. The energy consumptions of the AAT and Xiao’s schemes are between the static and the ideal method. Besides, the energy consumption of AAT is always below that of Xiao’s scheme, and the gap becomes bigger with the increase of Rx sensitivity.

**Figure 8:**
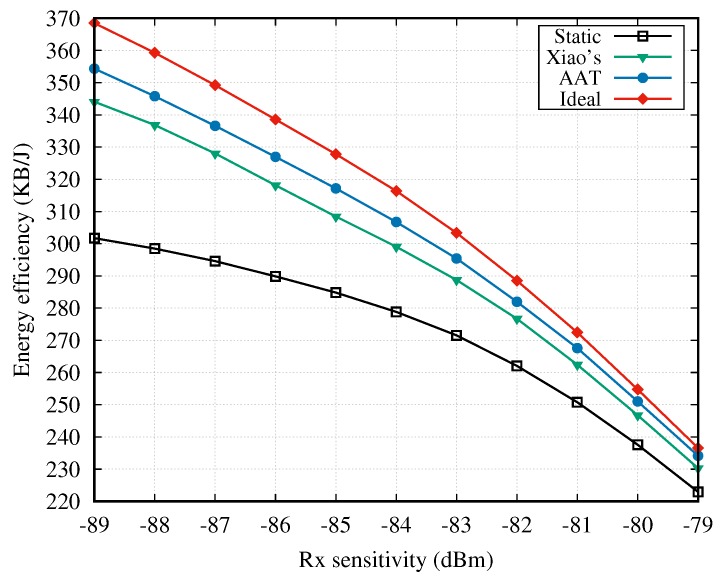
Energy efficiency vs. Rx sensitivity.

In Figure 8, we provide the simulation results in terms of energy efficiency, which jointly considers the throughput and the consumed energy. Clearly, the ideal and static methods achieve the best and worst energy efficiencies, respectively. The performances of AAT and Xiao’s method lie between these, and AAT exhibits a significant improvement in comparison with Xiao’s method. Moreover, with the rise of Rx sensitivity, the energy efficiencies of the four scheme decrease, and the gaps between them become narrower. This is mainly because a higher Rx sensitivity leads to more packets loss, even though the maximal transmission power level is adopted.

**Figure 9:**
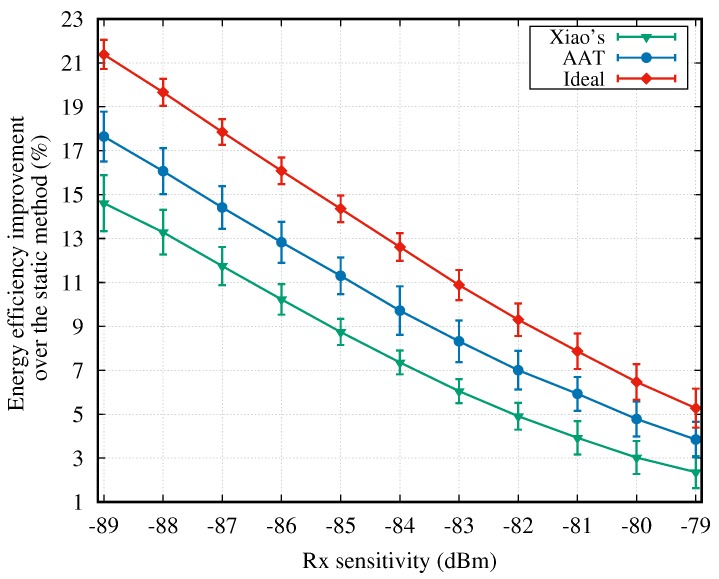
Energy efficiency improvement over the static scheme vs. Rx sensitivity.

All aforementioned figures are provided for the case in which only one channel dataset, i.e., $CD_{1}$, is imported. Simulations for other datasets are similar. Figure 9 shows the average energy efficiency improvement over the static method for the AAT and Xiao’s scheme. This figure shows the overall simulation results over all 16 channel datasets. Note that the energy efficiency improvement refers to the percentage improvement, compared to the static method. Clearly, AAT achieves a much better energy efficiency improvement (6.43–10.28%) in comparison with the conventional Xiao’s method (0–2.89%). Figure 9 also shows that the energy efficiency advantage of AAT is more significant when the Rx sensitivity is lower.

#### 4.2.2. Adaptive Transmission with Cooperation

As cooperative transmission and NC technologies are considered as promising methods for increasing the transmission reliability, we evaluated the performances when two-hop cooperation is considered. Specifically, we provide the performance comparison when two types of cooperative mechanisms are adopted. The first cooperative mechanism does not use NC, and is called non-NC cooperative mechanism. The sensor node(s) predicted to be in outage in the current beacon period is (are) selected as the relayed node(s), and one relay node is chosen randomly from the other sensor nodes. The second cooperative mechanism (denoted as NC cooperative mechanism) explores the RLNC (Random Linear Network Coding) technology in the relay node. Specifically, the hub selects one relay node, which stays in the Rx state during the whole DTP period to receive the data packets from all other sensor nodes. Then, the relay node performs the RLNC operation on all received packets to generate NC packets. These NC packets are sent to the hub in RTP. An advantage of the NC cooperative mechanism is that the hub does not specify the relayed node(s), and data packets from all sensor nodes have the chance to be recovered by the successful two-hop cooperative transmission. For instance, if the sensor node transmits two packets during its SUI and the RTP is configured as two times of the length of one SUI, then up to four packets in the DTP can be recovered by the successful transmission of four NC packets. However, the NC cooperative mechanism requires the relay node to stay in the active state during the entire DTP period, which increases its energy consumption.

**Figure 10:**
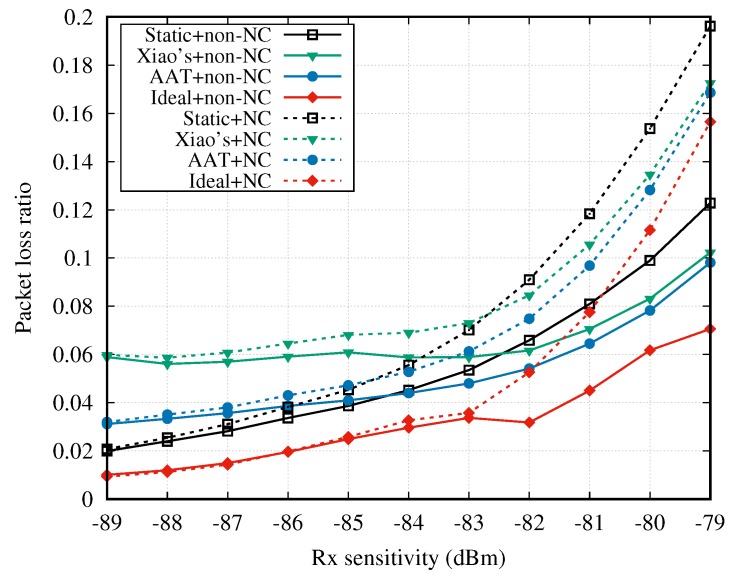
Packet loss ratio vs. Rx sensitivity.

By importing the channel dataset $CD_{1}$, Figure 10 shows the average PLR of sensor nodes with varying Rx sensitivity at the hub side. As expected, both non-NC and NC cooperative mechanisms significantly reduce the PLR in comparison with the case without cooperation (cf. Figure 6). In Figure 10, non-NC cooperative mechanism always has a better performance than the NC cooperative mechanism in terms of PLR. Moreover, the PLR gap between the curves becomes bigger with the increasing Rx sensitivity. Based on the simulation configurations listed in Table 3, the length of RTP is two times the length of one SUI, and one sensor node sends two packets in its SUI. Hence, the relay node transmits at most four native or NC packets to the hub during the RTP period. In the NC cooperative mechanisms, if more than four packets are lost in the DTP, no lost packets could be recovered because the degree of freedom is not sufficient [63]. Instead, in the non-NC cooperative mechanisms, the relay node could always recover the lost packets from the relayed node if the two-hop transmission is successful, regardless of the number of lost packets in one DTP. This is the main reason the gap between non-NC and NC cooperative mechanisms is widened when the Rx sensitivity is increased.

**Figure 11:**
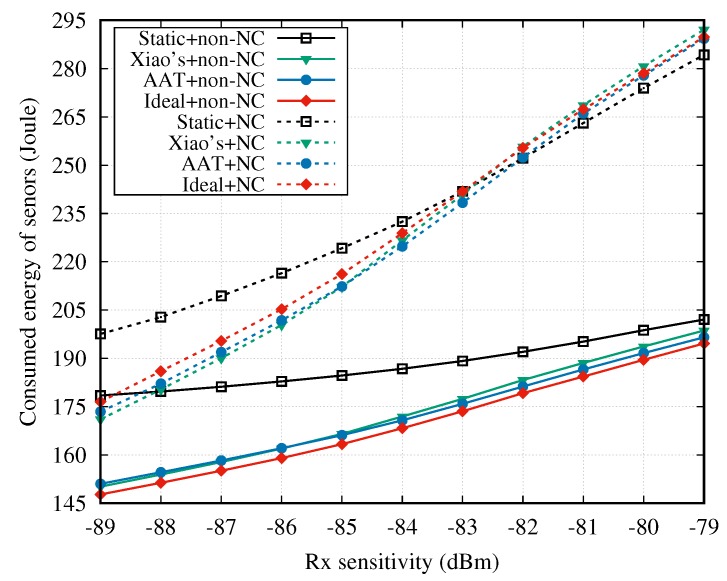
Energy consumption vs. Rx sensitivity.

Next, the energy consumption results are illustrated in Figure 11. As shown in this figure, the NC cooperative mechanisms consume much more energy compared to the non-NC cooperative mechanisms. The main reason is that, in the NC cooperative mechanisms, when the sensor is selected as the relay node, it stays in the Rx state during the entire DTP to receive the data packets from the remaining sensors. In contrast, the relay node in non-NC cooperative mechanisms only wakes up during the SUI(s) of the relayed node(s), which consumes less energy.

**Figure 12:**
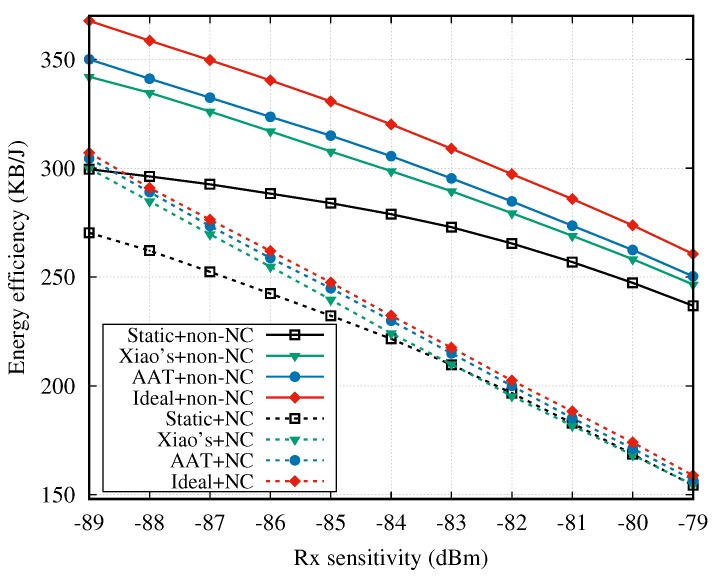
Energy efficiency vs. Rx sensitivity.

Figure 12 shows the energy efficiency when the two cooperative mechanisms are adopted. Clearly, compared to the NC cooperative mechanism, the non-NC method exhibits an advantage in terms of energy efficiency.

**Figure 13:**
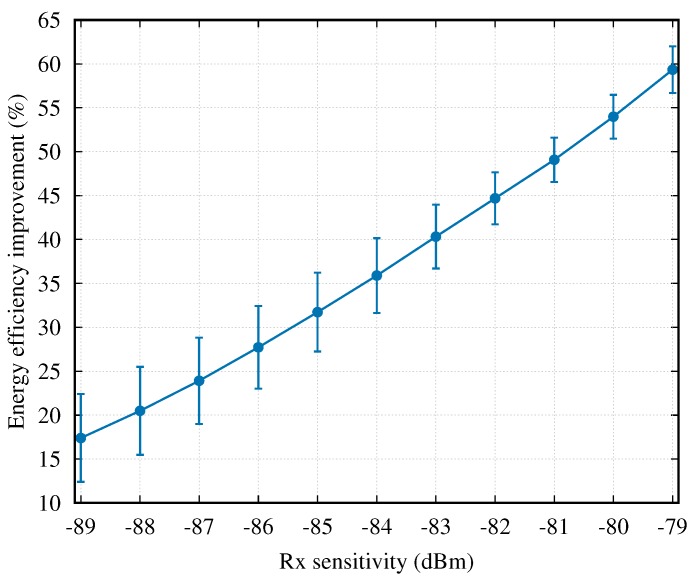
The energy efficiency improvement of the “AAT+non-NC” scheme over the “AAT+NC” scheme, when the Rx sensitivity varies.

Similar to the cases without two-hop cooperation, we also provide the overall energy efficiency results over all channel datasets collected from real WBAN scenarios, i.e., $CD_{1}$–$CD_{16}$. Figure 13 shows the average energy efficiency improvement of the “AAT+non-NC” scheme over the “AAT+NC” scheme. As shown in this figure, the non-NC cooperative mechanism achieves a much better energy efficiency in comparison with the NC cooperative mechanism for all collected channel datasets, and the improvement is enlarged (from 17.4% to 59.3%) with the increase of Rx sensitivity.

Based on the above simulation results, we have the following observations. Firstly, our newly proposed AAT scheme is capable of improving the transmission reliability and at the same time reducing the energy consumption. Compared to the Xiao’s TPC scheme, AAT improves the performance in terms of PLR, energy consumption, and energy efficiency. Secondly, we found that, in the star topology network, using NC technology to assist the uplink two-hop transmission may not be a good choice. The newly proposed non-NC cooperative mechanism achieves a better PLR performance while consuming less energy.

## 5. Conclusions

In this paper, we jointly consider transmission power control, dynamic slot scheduling and two-hop cooperative mechanism to achieve a better trade-off between transmission reliability and energy consumption. Motivated by the significant autocorrelation characteristic of on-body channels in the daily WBAN scenarios, we propose an Autocorrelation-based Adaptive Transmission (AAT) scheme that uses a temporal autocorrelation model to predict channel conditions. Then, the estimated channel conditions are used to optimize the transmission power level and the transmission order of all sensor nodes for the next superframe. AAT scheme is designed to be compatible with IEEE 802.15.6. We also evaluate the performance of the newly proposed scheme by importing the channel datasets collected from real WBAN daily scenarios into our simulation model. Simulation results demonstrate that the proposed method can effectively improve the transmission reliability while reducing the energy consumption. Moreover, we provide the performance evaluation when two-hop cooperative transmission is associated with the proposed AAT to further reduce the PLR. Two types of cooperative mechanisms are compared, i.e., the non-NC mechanism and the NC cooperative mechanism. Based on the evaluation results, the NC cooperative mechanism does not exhibit the advantage in all performance metrics, i.e., PLR, energy consumption, and energy efficiency. Hence, using NC technology to assist the uplink two-hop transmission may not be practical in the context of WBANs. 

## Figures and Tables

**Table 1:** The power consumptions in different states for CC2420.

State	Tx Power (dBm)	Current Consumption (mA)	Working Power (mW)
Rx	-	18.8	62
Sleep	-	0.42	1.4
Tx	0	17.4	57.42
	−1	16.7	55.18
	−3	15.3	50.69
	−5	14	46.2
	−7	12.8	42.24
	−10	11	36.3
	−15	9.9	32.67
	−25	8.5	29.04

**Table 2:** The transition delay and power consumption for CC2420.

From	To	Consumed Power (mW)	Delay (ms)
Sleep	Rx	62	0.194
Sleep	Tx	62	0.194
Rx	Sleep	1.4	0.05
Rx	Tx	62	0.01
Tx	Sleep	1.4	0.05
Tx	Rx	62	0.01

**Table 3:** Simulation parameters for AAT.

Parameter	Value
**Application Layer**	
PacketSize	105 bytes
PacketRate	25 Pkts/s
**Routing Layer**	
PacketOverhead	10 bytes
**MAC Layer**	
SuperframeLength	80 ms
SlotLength	5 ms
RAP1Length	10 ms
DTPLength	50 ms
RTPLength	20 ms
SUI	10 ms
pTIFS	0.03 ms
Data’s AckType	N-Ack
Control’s AckType	I-Ack
PacketOverheader	7 bytes
**Radio Layer**	
DataRate	250 kbps
ModulationType	PSK
Bandwidth	20 MHz
CarrierFreq	2400.0 MHz
NoiseFloor	−101 dBm
Rx_sensitivity	−89 dBm
CCAthreshold	−95 dBm
SymbolsForRSSI	8 bits
InitialTxPower	0 dBm
FrameOverheader	6 bytes
**others**	
SensorNumber	5
sensor’s initialEnergy	2430 J
Wireless Channel	TraceChannel
PLR_r	0.02
SimulationTime	3600 s

## Abbreviations

The following abbreviations are used in this manuscript:

AAT	Autocorrelation-based Adaptive Transmission
DSS	Dynamic Slot Scheduling
DTP	Direct Transmission Phase
IEEE	Institute of Electrical and Electronics Engineers
ISI	Inter-Symbol Interference (ISI)
ISM	Industrial, Scientific and Medical
LQI	Link Quality Indication
MAP	Managed Access Phases
N-Ack	No Acknowledgement
NC	Network Coding
pSIFS	Short Interframe Separation Time
QoS	Quality of Service
PLR	Packet Loss Ratio
RLNC	Random Linear Network Coding
RSSI	Received Signal Strength Indicator
RTP	Relay Transmission Phase
SAR	Specific Absorption Rate
SUI	Scheduled Uplink Interval
TAM	Temporal Autocorrelation Model
TDMA	Time-Division Multiple Access
TPC	Transmission Power Control
WBAN	Wireless Body Area Network
WSN	Wireless Sensor Network
WSS	Wide Sense Stationary
